# Herbal Formula Modified Bu-Shen-Huo-Xue Decoction Attenuates Intervertebral Disc Degeneration via Regulating Inflammation and Oxidative Stress

**DOI:** 10.1155/2022/4284893

**Published:** 2022-02-02

**Authors:** Jialiang Lin, Jionghui Gu, Dongwei Fan, Weishi Li

**Affiliations:** ^1^Department of Orthopaedics, Peking University Third Hospital, Beijing 100191, China; ^2^Beijing Key Laboratory of Spinal Disease Research, Beijing, China; ^3^Engineering Research Center of Bone and Joint Precision Medicine, Ministry of Education, Beijing, China; ^4^Department of Ultrasound, The First Affiliated Hospital, College of Medicine, Zhejiang University, Hangzhou 310003, China

## Abstract

**Objective:**

This study aims to clarify the potential mechanism of modified Bu-Shen-Huo-Xue decoction (MBSHXD) in treating intervertebral disc degeneration (IDD) with methods of network pharmacology and molecular docking.

**Methods:**

An MBSHXD and IDD-related common target gene set was established through TCMSP, UniProt, and two disease gene databases. GO and KEGG enrichment analysis and protein-protein interaction (PPI) networks were performed through the R platform and STRING to discover the potential mechanism. Molecular docking between the active ingredients and the core genes is used to calculate the binding energy.

**Results:**

A total of 147 active ingredients and 79 common genes (including 10 core genes, TNF, VEGFA, IL6, MAPK3, AKT1, MAPK8, TP53, JUN, MMP9, and CXCL8) were identified. The results of GO and KEGG enrichment analysis showed that MBSHXD plays an essential role in regulating inflammation and oxidative stress. The meaningful pathways are the AGE-RAGE signaling pathway in diabetic complications, the IL-17 signaling pathway, the TNF signaling pathway, the PI3K-Akt signaling pathway, the MAPK signaling pathway, and apoptosis. In addition, the PPI network and molecular docking further demonstrated the roles that nine bioactive ingredients of MBSHXD play in IDD treatment through their interference with core target proteins.

**Conclusion:**

This study reveals that MBSHXD has the characteristics of a “multi-component, multi-target, and multi-pathway” in the treatment of IDD by regulating inflammation and oxidative stress, and network pharmacology may provide a feasible method to verify the molecular mechanism of MBSHXD for IDD by combining with molecular docking.

## 1. Introduction

As a common symptom, low back pain is the leading cause of years living with disability [1]. It is reported that up to 40% of low back pain is associated with intervertebral disc degeneration (IDD) [2]. IDD is a highly prevalent degenerative spinal disorder that manifests as pain, numbness, and even paralysis of the lower limbs. The discomfort caused by IDD brings great pain and inconvenience to life and work of patients. However, except for nonsteroidal anti-inflammatory drugs (NSAIDs) to relieve acute pain, no effective drugs against IDD are currently available.

In recent years, traditional Chinese medicine (TCM), as an essential branch of complementary and alternative medicine, has received increasing attention for its role in various diseases [3–5]. Modified Bu-Shen-Huo-Xue decoction (MBSHXD), which adds or reduces several herbs based on Bu-Shen-Huo-Xue decoction, is a traditional Chinese herbal recipe that was widely used in elderly patients for fractures therapy in ancient times. MBSHXD consists of the following 9 medicinal herbs: *Radix Rehmanniae Preparata, Cortex Eucommiae, Radix Aconiti Lateralis Preparata, Fructus Lycii, Fructus Corni, Semen Persicae, Flos Carthami, Rhizoma Dioscoreae, and Radix Glycyrrhizae.* According to TCM, MBSHXD has the effects of nourishing the kidney, promoting blood circulation, and strengthening the bones. Recent studies have shown that MBSHXD may delay the progression of degenerative diseases of the musculoskeletal system, including osteoarthritis and IDD [6, 7]. Zhu et al. [7] reported that MBSHXD could inhibit the calcification of the cartilage endplate in gerbils during the process of aging. However, its underlying mechanisms are still unclear.

**Figure 1:**
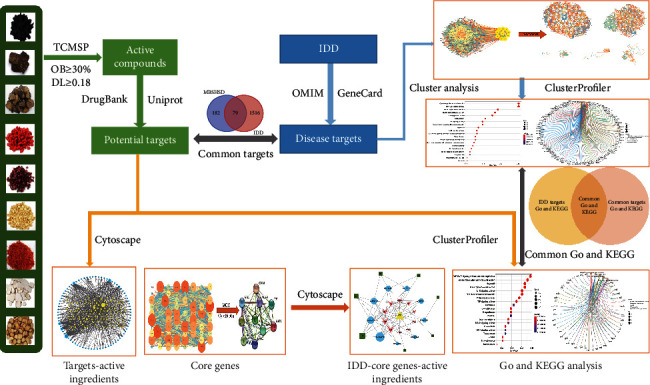
Flowchart to explore the possible mechanism of modified Bushen Huoxue decoction against intervertebral disc degeneration (IDD).

Network pharmacology, an emerging and interdisciplinary research approach, is based on traditional pharmacology, bioinformatics, chemoinformatics, and network biology [8, 9]. The development of network pharmacology promotes the transition from the era that a drug fits into the specific target to the generation of mapping the polypharmacology network onto the human disease-gene network [10–12]. Due to the complexity of its composition and function, the clinical application of TCM is distinctly hampered. Fortunately, network pharmacology could be a valuable tool for analyzing and constructing a “drug-target-disease” interaction network to reveal the molecular mechanisms of action between mutiherbs and the disease [13–15]. Recent studies have confirmed that network pharmacology can help develop TCM applications for the treatment of other diseases or the development of new drugs [16–19] and provide biochemical principles to manage current pandemic diseases, such as COVID-19 [20]. This study aims to clarify the potential mechanism of MBSHXD in the treatment of IDD. The flowchart of our research is shown in Figure 1.

## 2. Materials and Methods

### 2.1. Active Ingredients and Target Genes in MBSHXD

We got the active ingredients of each herb in MBSHXD through the Traditional Chinese Medicine Systems Pharmacology (TCMSP) database (https://tsmspw.com) [21]. The oral bioavailability (OB) ≥ 30% and drug-likeness (DL) ≥ 0.18 were set as filtration conditions. Next, the potential target proteins of the selected active ingredients were mined in the DrugBank database (http://www.drugbank.ca) [22].Then, we utilized the UniProt database (https://www.uniprot.org/) [23] to obtain the unique corresponding gene names and to establish the MBSHXD target gene set.

### 2.2. The Related Target of IDD

IDD-related targets were obtained through retrieving GeneCards (https://www.genecards.org) [24] and Online Mendelian Inheritance in Man (OMIM) (https://omim.org, update November 25, 2020) [25] using the keywords “intervertebral disc degeneration.” GeneCards is a comprehensive, user-friendly database that provides information of all annotated and predicted human genes, and we screened for targets with a relevance score ≥10 [26]. The OMIM database provides an organized description of human genes and phenotypes and the relationships between them and is a comprehensive, authoritative, and timely resource for research [25]. Finally, the results of the two databases were summarized, integrated, and deduplicated, and then 1595 potential targets were obtained, and all targets were standardized in the UniProt database (https://www.uniprot.org/) [23].

### 2.3. Network Visualization and Enrichment Analysis

A common gene set between MBSHXD targets and IDD-related genes was built by the Venn diagram. According to a common gene set, we identified the active components corresponding to each gene and constructed an “active ingredient-common target gene network” using Cytoscape 3.7.2 software [27]. In addition, we have established a “disease-core genes–active ingredients–herbs” network.

The biological functions of genes were revealed in three different aspects, including biological processes (BPs), cell components (CCs), and molecular functions (MFs), by gene ontology (GO) enrichment analysis. The Kyoto Encyclopedia of Genes and Genomes (KEGG) is a database resource for understanding high-level functions and utilities of the biological system, such as cells, organisms, and ecosystems, from molecular-level information. We used the clusterProfiler package of the R platform for GO and KEGG functional enrichment analysis [28].

### 2.4. Protein-Protein Interaction (PPI) Network Construction and Core Gene Selection

The common genes of MBSHXD targets and IDD-related genes were used to build a PPI network through the STRING database (https://www.string-db.org/) [29]. In this database, we set the organism as “*Homo sapiens*” and a confidence score >0.4. In the same way, the PPI network of IDD targets was also established. The PPI network of the common target was imported into the Cytoscape software to make it visible. A cytoHubba plugin [30] was used to identify the top 10 core genes by a method of maximal clique centrality (MCC). In addition, the cluster analysis was performed for the IDD targets PPI network by using the MCODE plugin in Cytoscape software [31]. The parameter conditions are as follows: a node score cutoff = 0.2, k core = 2, maximum depth = 100, and a degree cutoff = 2 [32].

### 2.5. Molecular Docking

The 3D structures of the proteins encoded by the core genes and the 2D structures of active ingredients were downloaded from the RSCB PDB database (https://www.rcsb.org/) and PubChem database (https://pubchem.ncbi.nlm.nih.gov/), respectively. The energy of the molecular ligand was optimized using the MM2 calculation method and derived using the ChemBio 3D software. The receptor protein was dehydrated and small-molecule ligand was removed with PyMOL 2.4.0 software. The AutoDock tool performed hydrogenation of proteins and active ingredients. Finally, AutoDock Vina was used to calculate the binding energy between the receptor protein and active components [33].

## 3. Result

### 3.1. Screening of Candidate Targets and Active Ingredients

**Figure 2:**
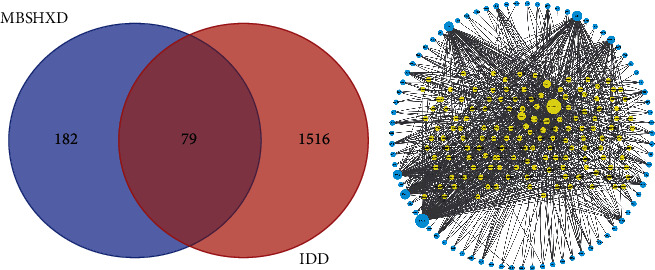
Construction of common gene-active ingredients network. (a) Venn diagram. (b) Common targets-active ingredients network. Yellow nodes represent the common targets of IDD and modified Bushen Huoxue decoction; blue nodes represent the active ingredients related to the common targets. The line between two nodes represents the interaction; the size of each node represents the number of connections.

The MBSHXD contained the following 9 main herbs: *Radix Rehmanniae Preparate* (Shudihuang), *Cortex Eucommiae* (Duzhong), *Radix Aconiti Lateralis Preparate* (Fuzi), *Fructus Lycii* (Gouqizi), *Fructus Corni* (Shanzhuyu), *Semen Persicae* (Taoren), *Flos Carthami* (Honghua), *Rhizoma Dioscoreae* (Shangyao), and *Radix Glycyrrhiza* (Ganzao). A total of 244 active ingredients of MBSHXD were obtained through searching TCMSP (Supplementary Table 1). Based on the active ingredients, we acquired 299 targets, which were protein names coded by genes (Supplementary Table 2). Then, after converting the gene symbols by the UniProt database, 261 drug targets were gained (Supplementary Table 3). In addition, a total of 1595 IDD-related genes were obtained from the two abovementioned disease gene databases. By intersecting the targets of MBSHXD and IDD, 79 common target genes were acquired, corresponding to which there were 147 active components (Supplementary Table 4, Figure 2(a)).

### 3.2. Common Targets-Active Ingredients Network

A network of common targets-active ingredients with 226 nodes and 904 edges was visualized using Cytoscape software (Figure 2(b)).

### 3.3. PPI Network Construction of IDD Target

**Figure 3:**
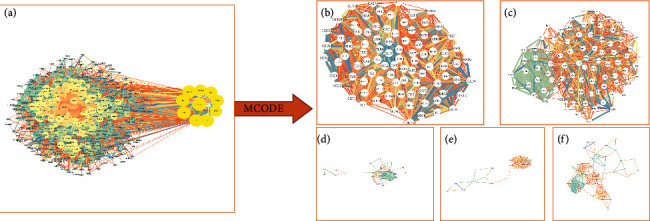
Protein-protein interaction (PPI) network and cluster analysis of the IDD targets. (a) PPI network of IDD targets. The 10 genes with the largest degree values are shown on the right with yellow nodes. (b–f) The top 5 cluster graphs of the IDD targets from its PPI network.

**Figure 4:**
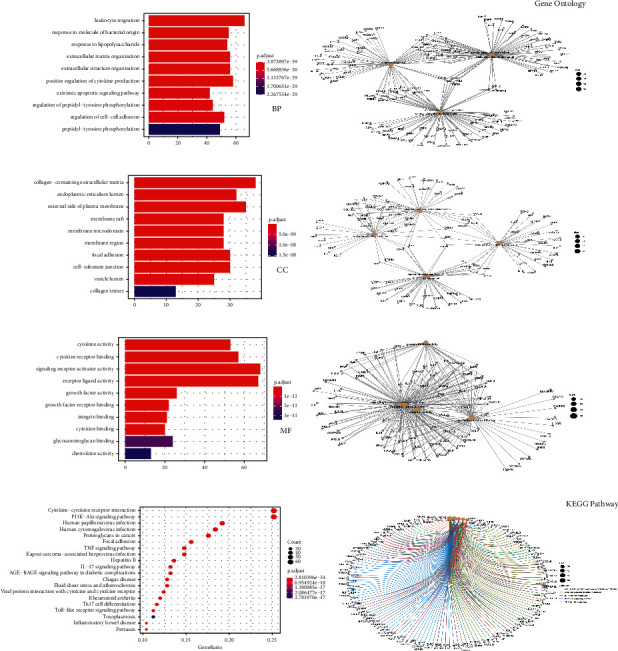
GO (including BP, MF, and CC) and KEGG analysis of IDD-related genes. (a, c, d) The top 10 significantly GO (BP, MF, and CC) enriched terms are arranged according to the adjusted *P* value. (b, e, f) Subnetwork showing the top five terms and related genes. (g) The 20 pathways with the lowest adjusted *P* values. The darker the color, the smaller the adjusted *P* value. The larger the circle, the greater the number of target genes in the term. (h) Subnetwork showing the top five KEGG pathways and related genes.

A protein-protein interaction network was established using the STRING database, which showed complex relationships between the proteins encoded by IDD genes (Figure 3). We selected 10 genes (AKT1, IL6, TP53, VEGFA, TNF, FN1, EGFR, EGF, MAPK3, and MYC) whose largest degree was demonstrated on the right. The PPI network was imported into the Cytoscape software for further cluster analysis and visualization with the MCODE plugin. Finally, the top five clusters were selected from 32 clusters based on their MCODE score (Figure 3 and Table 1). In addition, we performed GO functional enrichment and KEGG pathway analysis on the IDD target genes contained in these 5 clusters. Among the 291 genes, we acquired 3088 BPs, 93 CCs, 135 MFs, and 151 KEGG pathways with *P* value less than or equal to 0.05 (Supplementary Table 5). Then, Figure 4 shows the top 10 for GO functional enrichment and the top 20 for KEGG pathway analysis.

The biological processes of IDD may be mainly related to the inflammatory response and the exogenous pathways of apoptosis, such as leukocyte migration, extracellular matrix (ECM) organization, extracellular structure organization, and extrinsic apoptotic signaling pathways. The molecular functions of IDD would be related to cytokine activity, cytokine receptor binding, and signaling receptor activator activity. The cellular components associated with IDD might activate the collagen-containing ECM, endoplasmic reticulum lumen, and external side of the plasma membrane (Figure 4). Meanwhile, the results of KEGG indicated that the main pathways were related to cytokine-cytokine receptor interaction, the PI3K-Akt signaling pathway, and the TNF signaling pathway (Figure 4).

### 3.4. Enrichment Analysis of Common Genes

**Figure 5:**
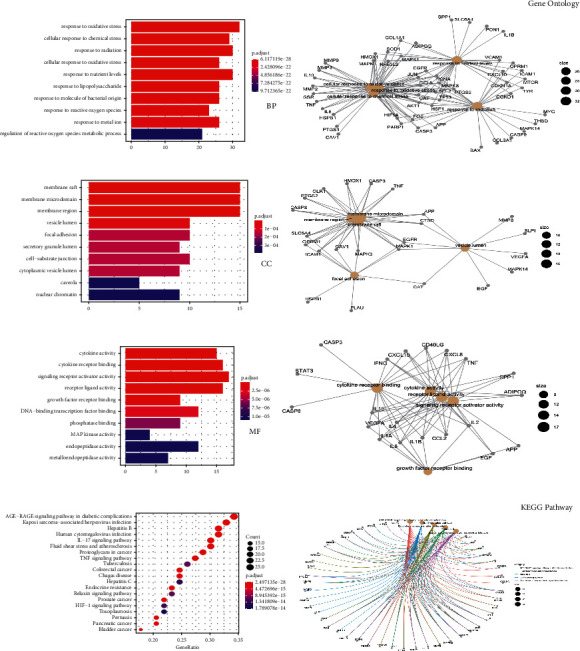
GO (including BP, MF, and CC) and KEGG analysis of common genes. (a, c, d) The top 10 significantly GO (BP, MF, and CC) enriched terms are arranged according to the adjusted *P* value. (b, e, f) Subnetwork showing the top five terms and related genes. (g) The 20 pathways with the lowest adjusted *P* values. The darker the color, the smaller the adjusted *P* value. The larger the circle, the greater the number of target genes in the term. (h) Subnetwork showing the top five KEGG pathways and related genes.

To further explore the interactions between common target genes and the mechanisms by which MBSHXD may treat IDD, GO enrichment analysis and KEGG pathway enrichment were performed using the R platform. We acquired a total of 2114 entries about biological processes. The top 10 are shown in Figure 5, among which 4 items were related to oxidative stress. By analyzing the results of the cellular component, we found that the target genes mainly acted in the membrane raft, membrane microdomain, and membrane region. The main molecular functions of common targets were cytokine activity, cytokine receptor binding, signaling receptor activator activity, and receptor-ligand activity. Simultaneously, the top 20 pathways were selected to investigate the possible mechanism of MBSHXD on the disease (Figure 5). The most meaningful pathways of common targets are the AGE-RAGE signaling pathway in diabetic complications, the TNF signaling pathway, and apoptosis. All the results of the enrichment analysis are listed in Supplementary Table 6.

### 3.5. PPI Network of Common Target and Core Genes

**Figure 6:**
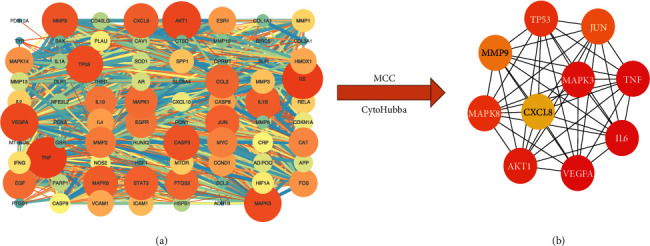
Confirmation of core genes. (a) Visualization of seventy-nine common targets protein-protein interaction (PPI) network with 79 nodes and 1487 edges. (b) PPI network of the core genes.

Firstly, we inputted 79 common genes into the STRING to construct the PPI network (Figure 6). To further obtain the core genes of the MBSHXD treatment for IDD, the obtained PPI network was imported into the software through the cytoHubba plugin with the MCC method. Finally, the top 10 genes were identified as core genes (TNF, VEGFA, IL6, MAPK3, AKT1, MAPK8, TP53, JUN, MMP9, and CXCL8). Fortunately, there are six genes (TNF, VEGFA, IL6, MAPK3, AKT1, and TP53) that overlap with the critical genes for IDD. In addition, we found that the PPI network for 10 core genes all had a node degree of 9 (Figure 6).

### 3.6. Selection of Critical Enrichment Analysis

After comparing and analyzing the KEGG enrichment of the disease targets and common targets of MBSHXD and IDD, we found 138 overlapping KEGG pathways (Supplementary Table 7). To further screen these pathways, we sorted them according to the proportion of genes and compared the potentially related IDD pathways in PubMed with the abovementioned ways. Finally, 9 relatively related IDD pathways were obtained.  Table 2 shows the basic information of the 9 pathways that may be relevant for IDD.

### 3.7. Disease-Core Genes-Active Ingredients-Herbs Network

**Figure 7:**
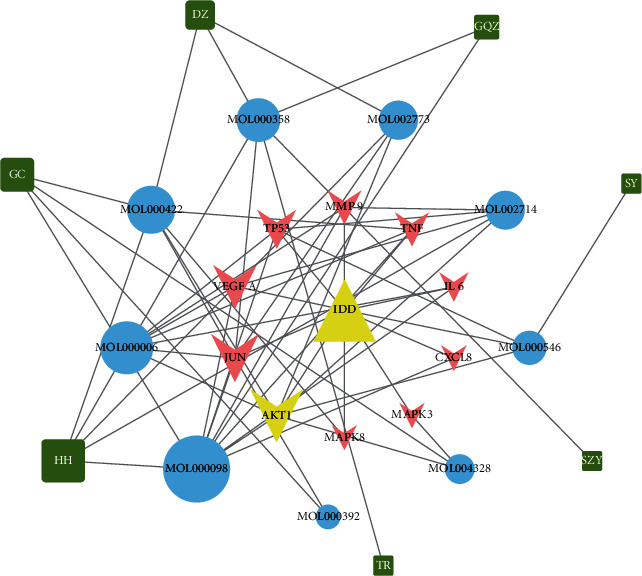
IDD-core gene-active ingredient-herb network. Red triangle nodes represent disease, pink arrow-like nodes represent core genes, blue circle nodes represent the active ingredients related to the core genes, and the green square nodes represent herbs. The size of each node was set according to its degree value.

According to the 10 core genes, we found the corresponding seven herbs and nine active components. The disease-core genes-active ingredients-herbs network is demonstrated in Figure 7. The 9 active components are sorted by degree as follows: quercetin (MOL000098), luteolin (MOL000006), kaempferol (MOL000422), beta-sitosterol (MOL000358), baicalein (MOL002714), beta-carotene (MOL002773), diosgenin (MOL000546), naringenin (MOL004328), and formononetin (MOL000392). The basic information of the nine active components in MBSHXD is listed in Table 3. Quercetin (MOL000098) was the essential active compound with the largest degree, which may play a key role in IDD treatment by MBSHXD.

### 3.8. Molecular Docking Verified Active Ingredients and Core Genes Encoding Proteins

**Figure 8:**
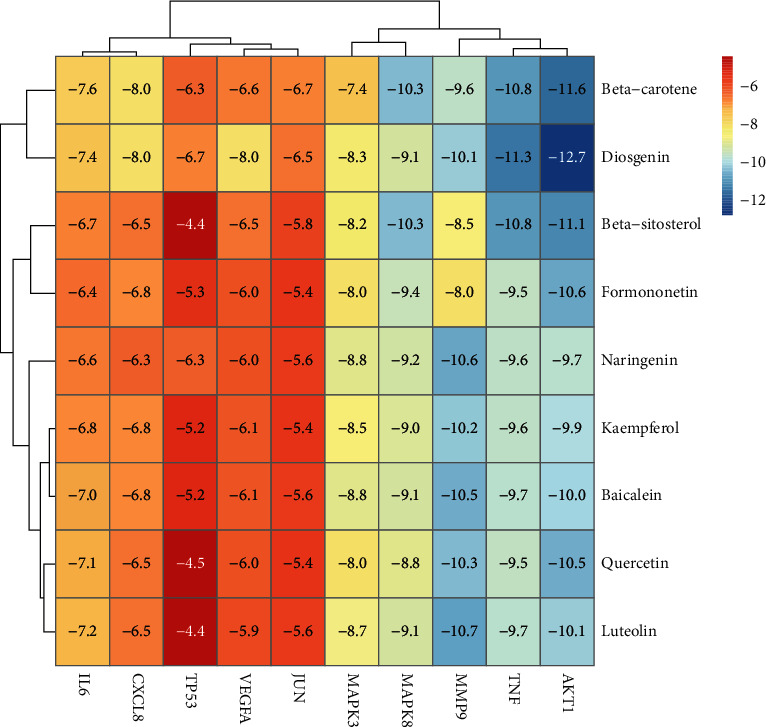
Heat map of molecular docking between 10 core genes and 9 active ingredients [34].

**Figure 9:**
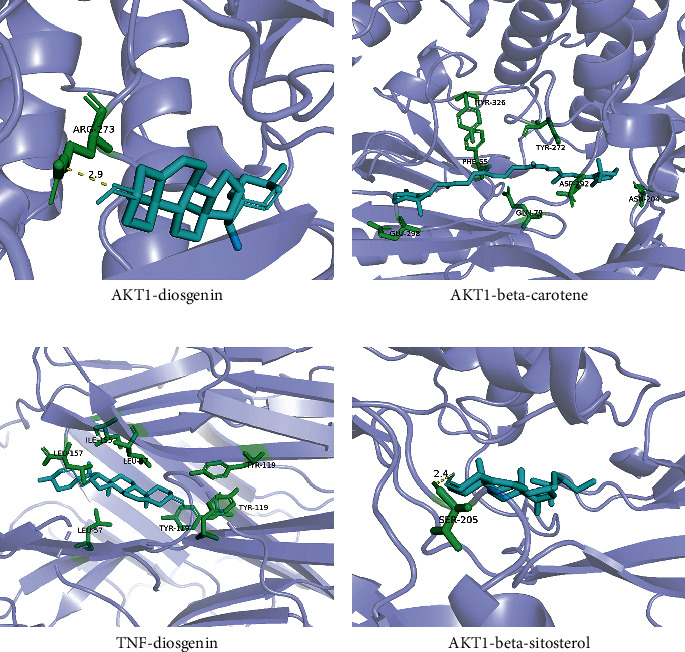
Molecular docking of “bioactive compound-hub gene.” (a) AKT1 to Diosgenin; (c) TNF to Diosgenin; (b) AKT1 to betacarotene; and (d) AKT1 to betasitosterol.

Ten key genes were used as receptor proteins, and nine active compounds were used as ligands for molecular docking verification. The details of 10 receptor proteins are listed in Table 4, and Figure 8 shows these results [34]. Binding energy, which is the criterion to judge stability, less than −5.0 kcal/mol was considered relatively stable between proteins and small-molecule compounds. As we expected, the binding energies between the active ingredients and the core genes were all less than −5.0 kcal/mol, indicating that all active compounds can easily enter the active pocket of proteins coded by core genes and bind stably. We selected 4 proteins and small molecules with the highest binding energy for demonstration (Figure 9).

## 4. Discussion

IDD is a widespread degenerative disease of the spine. With the increasing aging of society, the incidence of IDD has been on the rise in recent years and tends to be younger. The incidence rate of cervical, thoracic, and lumbar diseases caused by IDD is up to 80%, which has become a major global health problem [35]. At present, the interventions for IDD mainly include the use of pain relievers or surgical treatment [36, 37]. However, there is still a lack of effective nonsurgical treatments for IDD, making the exploration of novel and effective nonsurgical treatments for IDD a focus of research in degenerative spinal disorders. Notably, TCM has been used for more than 2,000 years to treat various diseases, including IDD [38]. TCM mainly focuses on expelling wind and cold, tonifying the liver and kidney in the treatment of IDD, supplemented by tonifying the spleen and replenishing qi, promoting blood circulation, and dredging collaterals [39–41]. MBSHXD, which comes from the ancient Chinese literature “Shangke Dacheng,” consists of 9 Chinese herbs, which are as follows: Shudihuang, Duzhong, Fuzi, Gouqi, Shanzhuyu, Taoren, Honghua, Shanyao, and Gancao [42]. It was demonstrated that MBSHXD could promote the proliferation of nucleus pulposus (NP) cells and remodel the ECM during IDD [43]. However, due to the complex composition and comprehensive action of MBSHXD, the specific mechanism of its treatment for IDD is still unclear, so we decided to use an approach of network pharmacology to reveal it and lay a foundation for further studies.

We used Cytoscape software to reconstruct the PPI network of common genes obtained from the STRING database and got 10 core genes (TNF, VEGFA, IL6, MAPK3, AKT1, MAPK8, TP53, JUN, MMP9, and CXCL8) of MBSHXD against IDD by the MCC algorithm. These core target proteins are involved in multiple signaling pathways such as inflammation, immunity, metabolism, and proliferation-related signaling pathways. Studies have confirmed that the process of IDD can be delayed by inhibiting the TNF-*α*-induced inflammatory response and reducing the expression of inflammatory cytokines, including IL-6 [44, 45]. It is worth noting that TP53 was found to be potentially involved in the progression of IDD by analyzing the microarray datasets of GSE19943, GSE15227, and GSE34095 [46]. Akt1 is a serine/threonine protein kinase that is involved in inflammation and cell metabolism through a wide variety of signaling pathways such as PI3K-Akt and MAPK signaling. Wang et al. found that the degeneration of NP cells was ameliorated by regulating the ITGA2/PI3K/Akt signaling pathway [47]. Moreover, Zhan et al. found that the degree of intervertebral disc degeneration was related to the loss of vascular buds and the downregulation of VEGFA and its receptors [48].

According to our “disease-core genes–active ingredients–herbs” network, nine bioactive compounds between the 10 core genes and the corresponding active compounds in MBSHXD were identified. Furthermore, three hub ingredients, quercetin, luteolin, and kaempferol, were selected, and they targeted the largest number of core genes. In vitro experiments showed that quercetin ameliorated the progression of IDD by suppressing the expression of senescence associated secreted phenotype factors and improving the senescence phenotype of NP cells via the Nrf2/NF-*κ*B axis [49]; luteolin, a natural flavonoid, has anti-inflammatory and anticatabolic effects [50]; kaempferol has been reported to regulate osteogenesis/adipogenesis balance and inhibit inflammation in bone mesenchymal stem cells to slow the progression of IDD [51]. These compounds are the material basis of MBSHXD in the treatment of IDD.

Based on the ten core genes and their corresponding core compounds, molecular docking was performed to demonstrate the binding energy between proteins and small-molecule compounds, which all showed good binding affinity. The abovementioned molecular docking results were presented in the form of a heat map by using R software. In addition, the software package ClusterProfiler was used for enrichment analysis of GO and KEGG pathways. The results of two enrichment analyses of GO and KEGG were compared with the IDD-related pathways searched in PubMed. We speculated that the therapeutic effect of MBSHXD in IDD may be related to its anti-inflammatory and antioxidative stress effects, which inhibit apoptosis and senescence of NP cells. Indeed, inflammation, ECM degradation, and senescence of NP cells are thought to be the main pathogenic mechanisms of IDD [52, 53]. The abovementioned mechanisms mainly involve the AGE-RAGE signaling pathway in diabetic complications, the IL-17 signaling pathway, the TNF signaling pathway, the PI3K-Akt signaling pathway, the MAPK signaling pathway, and apoptosis. It has been reported that the PI3K-Akt pathway is involved in the proliferation, apoptosis, senescence, and ECM metabolism of NP cells and is significantly associated with NP degeneration. It is considered as an essential signaling pathway involved in IDD [54–56]. TNF and IL-17 pathways play a synergistic role in IDD progression, mainly by promoting the release of inflammatory mediators, the apoptosis of NP cells, and the degradation of ECM [57–59]. In addition, the MAPK pathway is thought to primarily mediate the inflammatory response of NP cells to TNF-*α* stimulation [60]. Illien–Jünger et al. [61] suggested AGE accumulation is related to endochondral ossification, which may induce hypertrophy and osteogenic differentiation of intervertebral disc cells through the AGE/RAGE axis. More importantly, the accumulation of AGEs is closely associated with oxidative stress, which may lead to alterations in the oxidative microenvironment of NP and, in turn, contribute to IDD. Thus, targeted clearance of AGEs may be a promising direction to noninvasively slow down the progression of IDD.

However, our study also has some limitations. For example, the results of this study lacked validation by in vitro experiments, while further external validation in animals should be performed. In addition, the database we selected may not be comprehensive enough, and the active ingredients and related genes screened through the database may have been missed.

In conclusion, we reveal that MBSHXD has the characteristics of “multi-component, multi-target, and multi-pathway” in IDD treatment based on network pharmacology and molecular docking. MBSHXD retards the progression of IDD by regulating the antioxidant stress and inflammatory response, which inhibits apoptosis and senescence of NP cells. The primary mechanism is related to 9 core bioactive components, 10 core genes, and 9 related pathways. This study may inspire novel treatment strategies for IDD and inform future research.

## Figures and Tables

**Table 1:** The top 5 cluster information of the IDD targets from its network.

Cluster	Score	Nodes	Edges
1	61.506	90	2737
2	23.952	84	994
3	9.091	34	150
4	8.762	22	92
5	8.4	61	252

**Table 2:** The enriched 9 possible related pathways for IDD.

ID	Description	KEGG index enriched by IDD targets		KEGG index enriched by common targets		
		p. adjust	Count	p. adjust	Count	Count of active ingredients
Hsa04933	AGE-RAGE signaling pathway in diabetic complications	4.08*E* − 24	33	2.4971*E* − 28	25	125
Hsa04657	IL-17 signaling pathway	5.06*E* − 25	33	3.4116*E* − 24	22	243
Hsa04668	TNF signaling pathway	5.71*E* − 27	37	9.6621*E* − 20	20	245
Hsa04151	PI3K-Akt signaling pathway	1.19*E* − 29	63	7.355*E* − 10	19	47
Hsa04210	Apoptosis	1.32*E* − 11	24	2.0176*E* − 14	17	66
Hsa04010	MAPK signaling pathway	6.74*E* − 15	40	3.2112*E* − 10	16	54
Hsa05206	MicroRNAs in cancer	1.83*E* − 08	31	3.389*E* − 08	16	188
Hsa05010	Alzheimer's disease	2.69*E* − 06	30	3.3702*E* − 07	16	257
Hsa05215	Prostate cancer	6.98*E* − 17	26	2.1985*E* − 15	16	127

**Table 3:** Basic information of the nine active components in MBSHXD.

Molecule ID	Molecule name	PubChem CID	OB (%)	DL	Source (herb name)	Targeted core genes
MOL000098	Quercetin	5280343	46.43	0.28	Duzhong, Gouqizi, Honghua, Gancao	AKT1, CXCL8, IL6, JUN, MMP9, TNF, TP53, VEGFA
MOL000006	Luteolin	5280445	36.16	0.25	Honghua	TNF, MMP9, IL6, VEGFA, TP53, JUN, AKT1
MOL000422	Kaempferol	5280863	41.88	0.24	Duzhong, Honghua, Gancao	TNF, MAPK8, JUN, AKT1
MOL000358	Beta-sitosterol	222284	26.91	0.75	Duzhong, Gouqizi, Shanzhuyu, Taoren, Honghua	JUN
MOL002714	Baicalein	5281605	33.52	0.21	Honghua	MMP9, VEGFA, TP53, AKT1
MOL002773	Beta-carotene	5280489	37.18	0.58	Duzhong, Honghua	VEGFA, JUN, AKT1,
MOL000546	Diosgenin	99474	80.88	0.81	Shanyao	VEGFA, TP53, AKT1
MOL004328	Naringenin	932	59.29	0.21	Gancao	MAPK3, AKT1
MOL000392	Formononetin	5280378	69.67	0.21	Gancao	JUN

**Table 4:** Detailed information of the 10 core targets.

Protein	PDB ID	Relevant citation
TNF	7KP9	DOI: 10.1038/s41467-020-20828-3
VEGFA	4DEQ	DOI: 10.1074/jbc.M111.331140
ILA	1ALU	DOI: 10.1093/emboj/16.5.989
MAPK3	6GES	DOI: 10.1016/j.chembiol.2019.02.021
AKT1	6S9W	DOI: 10.1002/anie.201909857
MAPK8	4L7F	DOI: 10.1016/j.bmcl.2013.06.087
TP53	7DHZ	DOI: 10.1016/j.ccell.2020.11.013
JUN	1JUN	DOI: 10.1074/jbc.271.23.13663
MMP9	5CUH	DOI: 10.1016/j.ejmech.2016.01.053
CXCL8	1IKL	DOI: 10.1021/bi00040a008

## Data Availability

The data used to support the findings of this study are included within the article.

## Supplementary Materials

Supplementary Materials Supplementary Table 1: ingredients of each herb contained in MBSHXD. Supplementary Table 2: known therapeutic targets correspond to the active ingredients. Supplementary Table 3: the target protein corresponds to the gene name from UniPort. Supplementary Table 4: the common targets and corresponding active components. Supplementary Table 5: detailed information of GO and KEGG enrichment analysis for IDD-related targets. Supplementary Table 6: detailed information of GO and KEGG enrichment analysis for common targets. Supplementary Table 7: detailed information of overlapping KEGG pathways.
