# Supplementary figures and images for: Herbal Formula Modified Bu-Shen-Huo-Xue Decoction Attenuates Intervertebral Disc Degeneration via Regulating Inflammation and Oxidative Stress
Source: Evid Based Complement Alternat Med. 2022 Feb 2;2022:4284893. doi: 10.1155/2022/4284893 (PMC8828322; doi:10.1155/2022/4284893)

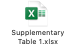

Supplement: Supplementary Materials — Supplementary Table 1: ingredients of each herb contained in MBSHXD. Supplementary Table 2: known therapeutic targets correspond to the active ingredients. Supplementary Table 3: the target protein corresponds to the gene name from UniPort. Supplementary Table 4: the common targets and corresponding active components. Supplementary Table 5: detailed information of GO and KEGG enrichment analysis for IDD-related targets. Supplementary Table 6: detailed information of GO and KEGG enrichment analysis for common targets. Supplementary Table 7: detailed information of overlapping KEGG pathways. [file 4284893.f1.zip › 4284893.f1/Supplementary Table 1 (1).docx]

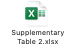

Supplement: Supplementary Materials — Supplementary Table 1: ingredients of each herb contained in MBSHXD. Supplementary Table 2: known therapeutic targets correspond to the active ingredients. Supplementary Table 3: the target protein corresponds to the gene name from UniPort. Supplementary Table 4: the common targets and corresponding active components. Supplementary Table 5: detailed information of GO and KEGG enrichment analysis for IDD-related targets. Supplementary Table 6: detailed information of GO and KEGG enrichment analysis for common targets. Supplementary Table 7: detailed information of overlapping KEGG pathways. [file 4284893.f1.zip › 4284893.f1/Supplementary Table 2 (1).docx]

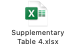

Supplement: Supplementary Materials — Supplementary Table 1: ingredients of each herb contained in MBSHXD. Supplementary Table 2: known therapeutic targets correspond to the active ingredients. Supplementary Table 3: the target protein corresponds to the gene name from UniPort. Supplementary Table 4: the common targets and corresponding active components. Supplementary Table 5: detailed information of GO and KEGG enrichment analysis for IDD-related targets. Supplementary Table 6: detailed information of GO and KEGG enrichment analysis for common targets. Supplementary Table 7: detailed information of overlapping KEGG pathways. [file 4284893.f1.zip › 4284893.f1/Supplementary Table 4 (1).docx]

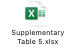

Supplement: Supplementary Materials — Supplementary Table 1: ingredients of each herb contained in MBSHXD. Supplementary Table 2: known therapeutic targets correspond to the active ingredients. Supplementary Table 3: the target protein corresponds to the gene name from UniPort. Supplementary Table 4: the common targets and corresponding active components. Supplementary Table 5: detailed information of GO and KEGG enrichment analysis for IDD-related targets. Supplementary Table 6: detailed information of GO and KEGG enrichment analysis for common targets. Supplementary Table 7: detailed information of overlapping KEGG pathways. [file 4284893.f1.zip › 4284893.f1/Supplementary Table 5 (1).docx]

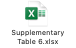

Supplement: Supplementary Materials — Supplementary Table 1: ingredients of each herb contained in MBSHXD. Supplementary Table 2: known therapeutic targets correspond to the active ingredients. Supplementary Table 3: the target protein corresponds to the gene name from UniPort. Supplementary Table 4: the common targets and corresponding active components. Supplementary Table 5: detailed information of GO and KEGG enrichment analysis for IDD-related targets. Supplementary Table 6: detailed information of GO and KEGG enrichment analysis for common targets. Supplementary Table 7: detailed information of overlapping KEGG pathways. [file 4284893.f1.zip › 4284893.f1/Supplementary Table 6 (1).docx]

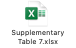

Supplement: Supplementary Materials — Supplementary Table 1: ingredients of each herb contained in MBSHXD. Supplementary Table 2: known therapeutic targets correspond to the active ingredients. Supplementary Table 3: the target protein corresponds to the gene name from UniPort. Supplementary Table 4: the common targets and corresponding active components. Supplementary Table 5: detailed information of GO and KEGG enrichment analysis for IDD-related targets. Supplementary Table 6: detailed information of GO and KEGG enrichment analysis for common targets. Supplementary Table 7: detailed information of overlapping KEGG pathways. [file 4284893.f1.zip › 4284893.f1/Supplementary Table 7 (1).docx]
